# Effect of a Garlic and Citrus Extract Supplement on the Lactation Performance and Carbon Footprint of Dairy Cows under Grazing Conditions in Chile

**DOI:** 10.3390/ani14010165

**Published:** 2024-01-04

**Authors:** Ruchita Khurana, Saheed A. Salami, Roberto Bergmann Poblete, Angela Fischer, Lisseth Aravena Cofré, Viviana Bustos, Bart M. Tas

**Affiliations:** 1 Mootral GmbH, D-13467 Berlin, Germany; 2 Mootral Ltd., Roseheyworth Business Park North, Abertillery NP13 1SX, UK; ssalami@mootral.com (S.A.S.); btas@mootral.com (B.M.T.); 3 Laboratorio de Carbono y Cambio Climático, Departamento de Acuicultura y Recursos Agroalimentarios, Universidad de Los Lagos, Avenida Fuchslocher #1305, Casilla 933, Osorno 5290000, Chile; robertoignacio.bergmann@alumnos.ulagos.cl (R.B.P.); angelaantonia.manriquez@alumnos.ulagos.cl (A.F.); lisseth.aravena@ulagos.cl (L.A.C.)

**Keywords:** garlic and citrus extract, milk yield, energy-corrected milk, dairy cows, grazing system, carbon footprint, life cycle assessment, Chile

## Abstract

**Simple Summary:**

There is increasing evidence that feeding a garlic and citrus extract supplement (GCE) to dairy cows could reduce enteric methane emissions and improve milk production. However, there is a lack of information on the effect of feeding with this supplement on the production performance of grazing cows. In a study conducted on a Chilean commercial farm, two experiments examined the impacts of feeding with GCE on the milk production performance and carbon footprint of grazing cows in the early- to mid-lactation and late-lactation stages. In both experiments, grazing cows were offered a supplementary concentrate without or with GCE (33 g/cow/d). Feeding with GCE increased feed intake and improved milk production, feed efficiency and lactation persistency in early- to mid-lactation and late-lactation grazing cows. Simulation of life cycle assessment indicated that the impacts of GCE on milk production efficiency resulted in a lower carbon footprint for milk. Thus, this study demonstrated that feeding with GCE could be a viable nutritional solution for improving sustainable dairy production in grazing systems.

**Abstract:**

Two trials were conducted to evaluate the effect of a garlic and citrus extract supplement (GCE) on the milk production performance and carbon footprint of grazing dairy cows in a Chilean commercial farm. A total of 36 early- to mid-lactation and 54 late-lactation Irish Holstein-Friesian cows were used in Trial 1 and Trial 2, respectively. In both trials, the cows were reared under grazing conditions and offered a supplementary concentrate without or with GCE (33 g/cow/d) for 12 weeks. The concentrate was fed in the afternoon when the cows visited the milking parlour. Consequently, the results of milk production performance in these trials were used to determine the effect of feeding with GCE on the carbon footprint (CFP) of milk using a life cycle assessment (LCA) model. In Trial 1 and Trial 2, feeding with GCE increased estimated dry matter intake (DMI, kg/d) by 8.15% (18.4 vs. 19.9) and 15.3% (15.0 vs. 17.3), energy-corrected milk (ECM, kg/d) by 11.4% (24.5 vs. 27.3) and 33.5% (15.5 vs. 20.7), and feed efficiency (ECM/DMI) by 3.03% (1.32 vs. 1.36) and 17.8% (1.01 vs. 1.19), respectively. The LCA revealed that feeding with GCE reduced the emission intensity of milk by 8.39% (1.55 vs. 1.42 kg CO_2_-eq/kg ECM). Overall, these results indicate that feeding with GCE improved the production performance and CFP of grazing cows under the conditions of the current trials.

## 1. Introduction

Chilean pastures used for dairy farming comprise around 1.5 million hectares and these grasslands provide feed for approximately 500,000 dairy cows [1,2]. Chilean milk production has been growing since 1985 to date, and this trend has been possible through increased pasture productivity, reseeding, fertiliser applications, rotational grazing and increased pasture utilisation [3,4,5]. The Los Lagos (41°28′18″ S 72°56′12″ W) and Los Ríos (48′30″ S 73°14′30″ W) regions in southern Chile account for 70% of the 2218 million litres of dairy production in the country [6]. In both regions, the total area of grassland consists of 50% naturalised pastures, 40% improved pastures and 10% sown pastures [7]. Direct grazing of permanent pastures is the most used system for milk production in southern Chile [8]. In Chilean grasslands, the predominant composition is 80–90% ryegrass (*Lolium perenne*) and 20–15% legumes (*Trifolium repens* and *pratense*) [7].

In dairy production, the improvement of milk production efficiency has significant environmental and economic implications. As milk production increases, the fixed costs on a dairy farm decrease relative to milk output thereby enhancing the labor and capital efficiency of housing and feeding [9]. Furthermore, the increasing pressure of environmental regulations has amplified the need to improve animal productivity as an effective strategy to reduce the carbon footprint (CFP; the amount of greenhouse gas emissions per unit of product) and enhance the sustainability of dairy farming [9]. In Chile, raw milk-receiving plants pay dairy producers based on kg of milk solids (fat + protein) or a mix of milk solids and volume [10]. Therefore, feeding strategies that improve milk production performance and efficiency are viable options for Chilean dairy farmers to increase profitability and reduce the environmental impacts of dairy production while optimizing the use of forage resources.

Several studies have demonstrated the enteric methane mitigation effect of feeding a garlic and citrus extract (GCE) supplement to dairy cows, sheep and beef cattle [11,12,13,14]. Supplementing GCE in a total mixed ration (TMR) has been shown to increase the milk yield and feed efficiency of dairy cows [11], potentially through positive modulation of rumen fermentation, which increases the production of volatile fatty acids (VFA) [15]. A recent study demonstrated that GCE supplementation also influenced changes in rumen microbiota and fermentation which led to a decrease in the molar ratio of acetate to propionate. Moreover, GCE supplementation has been linked to a greater abundance of the *Succinivibrionaceae* family and an inhibitory effect on the methanogens *Methanobrevibacter* genus [14]. However, no studies have been conducted to examine the effect of GCE supplementation on the production performance of dairy cows in grassland-based systems. Thus, the objective of this study was to evaluate the effect of GCE supplementation on the production performance of grazing dairy cows in a Chilean commercial farm in early- to mid-lactation and in late-lactation during the pasture growing season. Additionally, the results of milk production performance were used to perform a simulation to determine the effect of feeding with GCE on the CFP of milk using a life cycle assessment (LCA) model.

## 2. Materials and Methods

### 2.1. Study Location, Cows and Treatments

This study was conducted at La Querencia—a commercial dairy farm—located in the province of Osorno, commune of Puyehue, Los Lagos Region, Northern Patagonia, Chile (GCS-WGS84: −72.59, −40.72). La Querencia is a seasonal dairy farm with 240 Irish Holstein-Friesian cows milked in a pasture-based system. The cows are dried off between May to July; calving is concentrated between July and September, and milk production starts during the last week of July. Cows were milked twice daily (0300 and 1500 h) and were routinely checked for health status by the farm veterinarian. Animals were managed according to the standard operating procedures for this farm. The use of cows and the experimental procedures in this study complied with the guidelines of Article IV of Law 20.380 on the Protection of Animals in Chile and were approved by the Institutional Animal Care Committee of the University of Los Lagos, Chile.

This study consisted of two 12-week trials; Trial 1 (*n* = 36) was conducted during the early- to mid-lactation period and Trial 2 (*n* = 54) was conducted during the late-lactation period in 2021. Trial 2 was preceded by a covariate period of one week to collect data on milk yield and composition. From a herd of 240 cows, healthy lactating cows of second to fourth parity (early- to mid-lactation; 3.78 ± 1.35; late-lactation; 2.98 ± 0.83) were randomly selected based on body weight (early- to mid-lactation: 482 ± 43.9 kg; late-lactation: 513 ± 33.6 kg), milk yield (early- to mid-lactation: 26.9 ± 4.84 kg/d; late-lactation: 21.3 ± 3.16 kg/d) and absence of mastitis in the last 12 months. The early- to mid-lactation cows were weighed every 15 days during the 12-week trial period while the late-lactation cows were weighed only once at the beginning of the trial.

In both trials, the two experimental treatments were the control diet (CTRL; no supplementation) and the GCE supplement (Mootral Ltd., Abertillery, UK) applied at 33 g/cow/d. The cows were subsequently assigned to two feeding treatments in a randomised block design in groups of 18 cows per treatment for Trial 1. Trial 2 followed a completely randomised design and 27 cows in each group were randomly assigned to either of the two treatment groups. Cows grazed a pasture during the day and night. Additionally, when the cows came in for milking in the afternoon (1500 h), they were offered supplementary rolled corn and hay (Trial 1) or ryegrass and barley silage (Trial 2) that was spread in the troughs across the aisles, through which cows queued to enter the milking parlour. The cows were fed *ad libitum* rolled corn and hay (Trial 1) and silage (Trial 2) until they entered the milking parlour. Moreover, 500 g of concentrates was fed to the cows in the milking parlour, after which they left for the pasture. The chemical composition of the pasture, rolled corn, hay, silage and concentrate feed are shown in Table 1 and Table 2. The ingredients and nutritional composition were formulated according to NRC according to the lactating stage of the cows and their milk production levels [16].

The GCE supplement consisted of garlic and citrus extract powder (80%); it contained 900 g/kg DM, 20 g/kg ash, 167 g/kg crude protein, 166 g/kg crude fat and 6 g/kg crude fibre on a DM basis. The GCE supplement was stored in a closed container at room temperature (20–25 °C) at the farm. The GCE supplement was fed top-dressed on the concentrate feed provided individually to cows in the milking parlour feeders during afternoon milking at 1500 h.

### 2.2. Feed Sampling and Analysis

Silage was sampled according to the open silage protocol of the reference laboratory, Cooprinsem (Osorno, Region de Los Lagos, Chile). In brief, samples were taken from the freshly cut or daytime silage. A pool of 5 samples was taken from different points of the silo and carefully mixed to homogenise. Each sample contained at least 500 g of silage. Each pool was stored in a clean, airtight plastic bag, correctly labelled at 4 °C and delivered to the laboratory for processing within 12 h.

The La Querencia dairy farm pastures are naturalised, improved grasslands, which are frequently fertilised and receive amendments to increase production, with an annual herbage yield of 8 to 10 tons DM/ha. The botanical composition of these pastures is similar to the natural grasslands in Chile, consisting of perennial grasses such as *Dactylis glomerata*, Holcus lanatus, Bromus valdivianus, Trifolium repens, Lolium perenne, and Anthoxanthum odoratum. However, the proportion of species of higher forage value, such as *Trifolium repens*, Lolium perenne and Dactylis glomerata, is higher in these pastures. The pastures were randomly sampled at different points following the protocol from the reference laboratory. In brief, the paddock was walked in a zig-zag pattern. Sub-samples were taken 10 m from trees, troughs and edges. Forage was cut 5 cm from the ground, taking special care not to contaminate the sample with soil. Each sample contained at least 500 g of pasture and was stored in a clean, airtight, correctly labelled plastic bag at 4 °C and delivered to the laboratory for processing within 12 h. Near-infrared spectroscopy analysis (NIRS FOSS Model DS 2500, Foss Electric, Eden Prairie, MN 55344, USA) was performed for the prediction of the chemical composition of feed sources obtained during the early- to mid-lactation trial [17]. For the late-lactation trial, wet chemical analysis was used to determine the nutritional composition of feed sources. Wet chemical analysis was used to determine dry matter, acid detergent fibre, neutral detergent fibre and crude fat (ether extraction) on a Tecator Soxtec System HT 1043 (Tecator, Foss NA 7682 Executive Drive, Eden Prairie, MN 55344, USA) [18,19]. Crude protein was determined using a Nitrogen Combustion Analyser Kit (Leco FP-528. Leco, 3000 Lakeview Avenue, St. Joseph, MI 49085, USA) [19]. The metabolizable energy and net energy for lactation were calculated using the OARDC summative energy equations described by Weiss et al. [20].

### 2.3. Milk Yield and Composition Analysis

The daily milk yield of each cow was recorded using Waikato Milking System meters (Waikato Milking Systems NZ Ltd., Hamilton, New Zealand). Milk samples were collected from two consecutive milkings in weeks 2, 4, 6, 8 and 10 during early- to mid-lactation, and weeks 3, 6, 9, 11 and 12 during late-lactation. Milk samples were stored at 4 °C in the presence of preservative (bronopol-B2). Milk fat and protein percentage, and urea were analyzed using MilkoScan model FT7 RM (Foss Electric, Eden Prairie, MN 55344, USA) and somatic cell count (SCC) was analysed with Fossomatic 7DC (Foss Electric, Eden Prairie, MN 55344, USA). Milk solids were analysed according to the infrared-MilkoScan method following ISO 9622/IDF 141:2013 [21] and milk urea following ISO 8196-2/IDF128-2:2009 [22], while the counting of somatic cells was done using the fluoro-opto-electron method according to ISO 13366-2/IDF 148-2:2006 [23].

### 2.4. Calculations

Somatic cell counts were log_10_ transformed to achieve an approximate normal distribution before statistical analysis. The individual dry matter intake (DMI) of the experimental animals was estimated based on metabolic weight, fat-corrected milk and days in milk for each cow according to Equation (1) [24].

(1) $$DMI\;\left(\frac{kg}{day}\right)=\left(0.4762*4\%\;fat-corrected\;milk\;\left(\frac{kg}{d}\right)+0.07219*metabolic\;live\;weight\right)*\;\left(1-e^{\left(-0.03202*\left(Days\;in\;milk+24.9576\pm 5.909\right)\right)}\right)$$

where fat-corrected milk (4%) = 0.4 × kg milk + 15 × fat content (in kg) and metabolic live weight = live weight ^(0.75)^.

Energy-corrected milk (ECM) was calculated following Equation (2) [25].

(2) $$ECM\;\left(\frac{kg}{day}\right)=\frac{Milk\;yield*\left((Fat\%*0.38\right)+\left(Protein\%*0.21\right)+1.05)}{3.28}$$

Lactation persistency was calculated according to Equation (3) [26].

(3) $$Persistency\;\%=\left[1-\frac{\left(Milk\;kg\;earlier\;test\;-\;Milk\;kg\;later\;test\right)\times \frac{30\;days}{days\;between\;tests}}{Milk\;kg\;earlier\;test}\;\right]\times 100$$

### 2.5. Simulation Modelling of the Carbon Footprint of Milk

Simulation modelling was conducted to evaluate the impacts of feeding GCE to grazing cows in early- to mid-lactation and late-lactation on the CFP of milk based on the results of both trials. The simulation was conducted using the online Global Livestock Environmental Assessment Model-interactive tool (GLEAM-*i*), which can be accessed from https://gleami.apps.fao.org/ (accessed on 23 May 2023). GLEAM is a global, spatially explicit modelling framework that simulates the interaction of activities and processes involved in livestock production and the environment using an LCA approach. The model architecture, methods and functionality have been described by MacLeod et al. [27]. Further information on the description of GLEAM, including modules, background data, assumptions, variables and equations is available from https://www.fao.org/gleam/resources/en/ (accessed on 23 May 2023). The model quantifies GHG emissions (CO_2_, N_2_O, and CH_4_) resulting from production of the main livestock commodities, such as milk and meat, from different animal production systems, and can operate at sub(national), regional and global scales. GLEAM-*i* consists of three modules (herd, feed and manure) for data input, representing the main livestock production stages, and one calculation module for total herd emissions and production data outputs. The model allows users to define the baseline conditions (initial system state to which the scenario conditions will be compared) and scenario conditions (situations where changes to the baseline conditions have been made) to simulate the impact of a mitigation intervention.

In this study, simulation in GLEAM-*i* was performed for the grassland-based dairy system in Chile. Only the herd module was modified to define the input data for the baseline (control) and GCE scenarios in this study. In the herd module, we modified the “animal numbers” and “production” data inputs. For animal numbers, the number of adult females (cows) was set to 1000 for both baseline and GCE scenarios. The data inputs of production parameters (milk yield, and milk fat and protein contents) were modified for the baseline and GCE scenarios. The full lactation cycle of a dairy cow comprises about 305 days, with 205 days as the early- to mid-lactation period and the following 100 days as the late-lactation period. Given this information, the results for the CTRL and GCE cows obtained from the two trials (early- to mid-lactation and late-lactation periods) reported herein were extrapolated to obtain the annual milk yield (6676.5 vs. 7700.0 kg/cow/year) and milk fat content (3.86 vs. 3.86%) and milk protein content (3.67 vs. 3.67%) for the baseline and GCE scenarios, respectively. Notably, the milk fat and protein contents were assumed to be similar for the baseline and the GCE scenarios because there were no significant differences in these parameters between the CTRL and GCE groups in Trial 1 or Trial 2.

For the calculation module, the GLEAM model estimates the main sources of GHG emissions from feed production, enteric methane, manure emissions and embedded energy. The GLEAM model calculates emission intensity as the GHG emissions per unit of product protein (kg CO_2_-eq/kg protein). However, we expressed the emission intensity in this study as the GHG emissions per unit of ECM (kg CO_2_-eq/kg ECM). The annual ECM values for the baseline and GCE scenarios (6690.8 vs. 7716.5 kg/cow/year) were calculated according to Equation (2) using the data inputs for milk yield and milk fat and protein contents. Emission intensity based on ECM was calculated by dividing the total GHG emissions output by the annual ECM produced. Considering that ECM corrects the milk yield for fat and protein content, it allows for comparing the CFP among dairy studies on a common energy and protein basis and is regarded as the most applied functional unit in dairy LCA studies [28].

### 2.6. Statistical Analysis

Data on milk production performance were analysed using Proc Mixed of SAS (version 9.4, SAS institute, Cary, NC, USA) with a model that included the fixed effects of treatment, the weeks of milk measurements and their interactions, and the random effects of cows to correct for the natural variation between the experimental animals. For the trial conducted during early- to mid-lactation, the cows were blocked on parity and milk yield, and the block was fitted into the statistical model. During the late-lactation trial, however, the milk yield and composition parameters from the covariate week before the start of the trial were used as covariates for analysing milk yield and composition data. The least-square means and standard errors of the means (SEM) are reported; the differences between treatments were considered significant when *p* < 0.05 and a trend was observed when 0.05 < *p* < 0.10.

## 3. Results and Discussion

### 3.1. Effect of GCE on Milk Yield and Composition and Lactation Persistency

**Figure 1:**
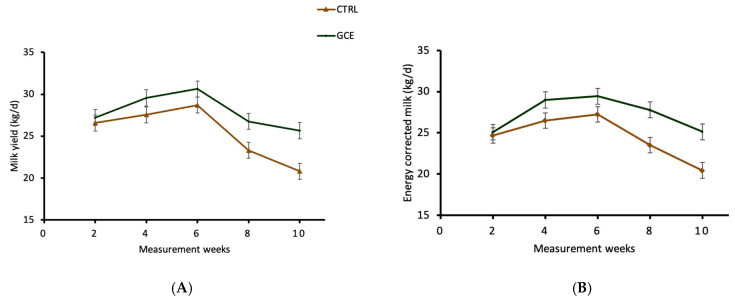
Weekly trends of (**A**) milk yield (kg/d) and (**B**) the energy-corrected milk of early- to mid-lactation cows in control and GCE-supplemented groups during Trial 1. Main effect of treatment: *p* < 0.001. Main effect of week: *p* < 0.001. Treatment × Week interactions: *p* > 0.05.

**Figure 2:**
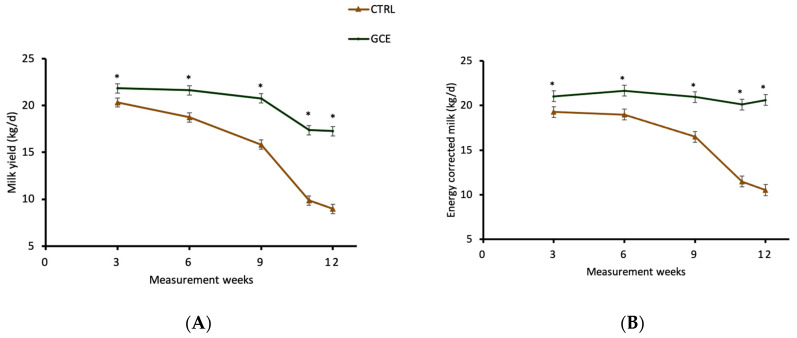
Weekly trends of (**A**) milk yield (kg/d) and (**B**) the energy-corrected milk of late-lactation cows in control and GCE-supplemented groups during Trial 2. Main effect of treatment: *p* < 0.001. Main effect of week: *p* < 0.001. Treatment × Week interactions: *p* < 0.001 (*).

Daily milk yield and ECM were significantly higher (*p* < 0.01) when GCE was supplemented in early- to mid-lactation (Table 3) and late-lactation cows (Table 4). The milk yield and ECM of GCE-fed cows increased by 2.6 kg/d (+10.2%) and 2.8 kg/d (+11.4%) during the early- to mid-lactation period compared with the CTRL cows, respectively (Table 3). In Trial 2, conducted during late-lactation, the milk yield and ECM of GCE-supplemented cows were significantly increased (*p* < 0.01) by 4.7 kg/d (+31.5%) and 5.2 kg/d (+33.5%), respectively, compared with the CTRL cows (Table 4). The treatment and week interaction was not significant for milk yield and ECM across Trial 1 (Figure 1A,B). However, the interaction between treatment and measurement week was significant for milk yield and ECM in late-lactating cows across the entirety of Trial 2 (Figure 2A,B). Moreover, milk protein and fat yields were increased (*p* < 0.01) by dietary GCE in both trials, mainly driven by the increased milk yield. However, there was no treatment effect (*p* > 0.05) on milk protein or fat percentages, urea concentration or SCC in early- to mid-lactation (Table 3) and late-lactation cows (Table 4).

In agreement with the current study, a 39.1% increase in daily milk yield was observed in late-lactation Holstein-Friesian cows fed TMR supplemented with a combination of garlic and minerals [29]. The positive response in milk production was attributed to efficient rumen fermentation caused by the garlic extract. Similarly, a significant 7.8% increase in milk yield was reported when GCE was supplemented in the TMR of the Holstein-Friesian dairy herd at a commercial farm in the United Kingdom [11]. In vitro and in vivo studies have demonstrated the potential of garlic products and citrus extracts to modulate the rumen microbiome and fermentation, particularly by increasing the production of total VFA, propionate and butyrate [15,30,31,32]. In a recently published study, feeding GCE tended to increase the proportion of propionate and reduce the acetate-to-propionate ratio, which was accompanied by changes in the rumen microbiota with a higher relative abundance of *Succinivibrionaceae* [14]. The potential effect of GCE to increase VFA production could enhance the metabolizable energy supply, resulting in improved milk production [33].

Furthermore, the presence of bioactive compounds (organosulfur compounds and flavonoids) in GCE could elicit functional effects such as antimicrobial, antioxidant, and immunomodulatory activities that could positively influence animal health and productivity [31,34]. Higher oxidative stress in cows has been linked to increased susceptibility to metabolic and reproductive disorders such as mastitis, metritis and the retention of foetal membranes in addition to other health problems [35]. Previous studies have shown that the negative effects of oxidative damage can be ameliorated with the inclusion of dietary antioxidants, which lead to peroxide scavenging thus reducing lipid peroxidation. This enhances dairy cows’ antioxidant status and improves their lactation performance [36]. Similarly, supplementing Holstein-Friesian cows in early lactation with a mixture of antioxidants and prebiotics increased their milk yield and protein content [37]. Additionally, supplementing silymarin extract (10 g/d) from the seeds of milk thistle (rich in flavonolignans) into the hay and silage-based diet of Italian Friesian dairy cows increased milk production by 7 to 13% within 7 to 30 days in milk [38]. The authors also noted that the treated cows attained their lactation peak before the control group and that high milk productivity remained persistent throughout lactation. This elucidates the positive effect of flavonoid-rich plant extracts on improving animal production. Thus, it can be postulated that the positive effects of GCE on increased milk yield could be partly attributed to improved rumen fermentation and the functional effects of the bioactive compounds on the health and metabolism of cows.

**Figure 3:**
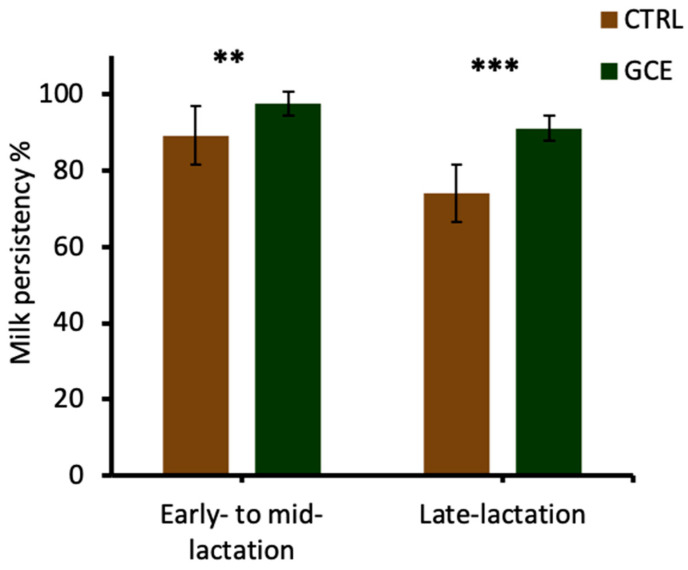
Lactation persistency of the control (CTRL) and GCE-supplementation groups in early- to mid-lactation and late-lactation trials. *** (*p* < 0.01), ** (*p* < 0.01).

In the present trials, the GCE-supplemented cows compared with the CTRL group displayed a higher milk production persistency of 97% vs. 89% during early- to mid-lactation (*p* < 0.05) and 91% vs. 74% during late-lactation (*p* < 0.01) (Figure 3). The ability of cows to sustain a high milk production rate for longer periods during lactation is an economically important trait for a profitable dairy enterprise. A significant effect of parity on lactation persistency has been reported, with cows in first parity displaying a higher lactation persistency compared with other cows [39]. The presence of a large number of secretory cells in the mammary glands of first-time lactating cows was cited as an explanation for their higher lactation persistency compared with subsequent lactations. This is because, in subsequent lactations, although increased milk secretion is observed by each secretory cell, the secretory activity of each cell is not maintained for as long a duration. This leads to a rapid reduction in secretory cells at an early stage of lactation, leading to the levelling-off of milk yield. In the present trial, none of the cows used in the trials were first parity, but feeding with GCE was able to maintain the secretory activity of the mammary cells during the early- to mid-lactation and late-lactation stages. As displayed in Figure 1 and Figure 2, the increased lactation persistency of GCE-supplemented cows implies that there was a slower rate of decline in milk yield during the trials. In comparison, the milk yield of cows in the CTRL group declined steadily during early- to mid-lactation and steeply during late-lactation following a typical lactation curve (Figure 1 and Figure 2).

### 3.2. Effect of GCE on Feed Intake and Feed Efficiency

Compared with the CTRL group, the estimated DMI of the GCE-fed cows was significantly higher (*p* < 0.01) by 8.15% and 15.3% for early- to mid-lactation and late-lactation cows, respectively (Table 3 and Table 4). However, the treatment × week interaction was significant only for the late-lactation trial. The observed trend of greater DMI can be attributed to the higher milk yield persistency of the GCE-supplemented cows, considering that the DMI was estimated based on fat-corrected milk, body weight and days in milk. The full dosage of 33 g GCE supplement mixed with the concentrate feed was well accepted by the cows, indicating that feed palatability was not affected. This observation is consistent with the lack of depression in feed intake found in other studies conducted with Holstein dairy heifers, cows or calves fed garlic cloves or powder at higher dosage rates of 7 or 10 g/kg of DMI, respectively [40,41]. In a study with multiparous mid-lactation Nordic Red cows, there was no effect of GCE supplement on feed intake when 22 g of GCE (44 g/d GCE) was fed mixed with 3 kg TMR in the morning and evening feeding [14].

Feed efficiency expressed as ECM/DMI was significantly higher (*p* < 0.01) for GCE-supplemented cows by 3.0% and 17.8% in early- to mid-lactation and late-lactation cows, respectively (Table 3 and Table 4). A tendency (*p* = 0.06) for greater milk yield/DMI (2.2%) was observed in early- to mid-lactation cows and milk yield/DMI was significantly higher by 16.5% (*p* < 0.01) in late-lactation cows fed GCE. Similarly, dietary GCE has been shown to improve the feed efficiency (milk yield/DMI) of lactating Jersey cows when supplemented in the TMR [11]. In another study, feeding garlic extract at 250 mg/kg body weight to Holstein cross-bred calves increased average body weight gain and feed conversion efficiency [42]. The significantly higher body weight gain observed in the supplemented calves was attributed to the functional antimicrobial and immunomodulatory properties of garlic compounds, which might have improved gut health and nutrient absorption in the young calves.

### 3.3. Effect of GCE on the Carbon Footprint of Milk

**Figure 4:**
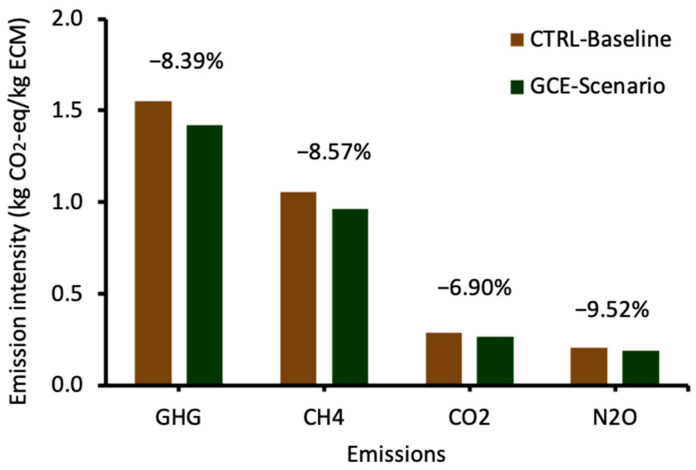
Emission intensity of milk, by emissions type, in the baseline (control) and GCE scenarios as simulated in the LCA model.

The LCA simulation results showed that feeding with GCE relatively reduced emission intensity (kg CO_2_-eq/kg ECM), presented as GHG, CH_4_, CO_2_ and N_2_O, by 8.39% (1.55 vs. 1.42), 8.57% (1.05 vs. 0.96), 6.90% (0.29 vs. 0.27) and 9.52% (0.21 vs. 0.19), respectively (Figure 4). The GHG emission intensity (i.e., CFP) found in the current study is close to the range of CFPs (1.54 to 3.57 kg CO_2_-eq/kg ECM) of Latin American dairy systems reported in a recent systematic review, with no significant differences between the CFPs of zero-grazing, semi-confinement and pasture systems [43]. However, another study observed a higher CFP (2.3 kg CO_2_-eq/kg milk) for a semi-intensive (based on 7 months in grazing and 5 months in confinement) dairy farm in Chile [44]. The greater production inputs and the associated emissions required for the semi-intensive system could partly explain the higher CFP compared with the full grazing system modelled in this study. Furthermore, the GHG emission intensity found in this study is considerably lower than the global average of 2.72 kg CO_2_-eq/kg ECM for grassland-based dairy systems. This suggests that the Chilean grazing system evaluated in this study is more efficient for milk production than the global average [45]. In comparison with other countries with predominantly pasture-based dairy systems, the CFP found in this study is relatively higher than the mean CFP values (0.75 to 1.20 kg CO_2_-eq/kg ECM) reported for Ireland [46,47,48], Australia [49,50], and New Zealand [51,52]. Nonetheless, it is noteworthy that the observed emission intensity reduction due to feeding with GCE could have a significant environmental impact. Considering that the GCE scenario reduced CFP by 0.13 kg CO_2_-eq/kg ECM compared with the CTRL baseline, this implies that feeding GCE to produce 1 million tonnes of ECM would potentially result in emission savings of 130,000 tonnes CO_2_-eq. In context, this GHG emission reduction is comparable to 27,837 intercontinental return trips between London and Santiago or the annual emissions of 85,209 cars.

Further analysis of the emission intensity by the sources of emissions, viz, enteric fermentation, manure, feed production and energy use, revealed that values of the GCE scenario were relatively lower compared with those of the CTRL baseline except for direct energy use CO_2_, which was unaffected (Table 5). This indicates that improving milk production efficiency is a viable strategy that could have a broad impact in reducing emission intensity from different emission sources such as enteric fermentation, feed, manure and energy use. In this study, enteric fermentation CH_4_ was the largest contributor to GHG emissions, accounting for about 66% of the total emissions. This is followed by emissions from feed and manure, accounting for 24% and 4% of the total emissions, respectively. In agreement with our results, enteric fermentation CH_4_ is generally the largest source of emissions in dairy production systems, followed by feed and manure emissions [53,54]. This emphasises the need to focus on enteric CH_4_ mitigation strategies to achieve significant emission reductions and more sustainable dairy farming [55]. It is noteworthy that the current simulation did not include the potential enteric CH_4_-mitigating effect of GCE [11,12,13,14], which could further reduce the GHG emission intensity of milk. Indeed, it would be interesting for future LCA studies to evaluate how the combined effect of GCE in improving milk production and enteric CH_4_ mitigation could influence the GHG emission intensity of dairy production. Moreover, future LCA work should include sensitivity analysis to evaluate how uncertainty factors related to varied production input parameters could influence the robustness of the current results.

## 4. Conclusions

Overall, the results from the present trials indicate that feeding GCE to grazing dairy cows improved the production performance of early- to mid-lactation and late-lactation cows, including increased milk yield and ECM, without affecting milk composition. Moreover, there was a concomitant improvement in lactation persistency and the feed efficiency of GCE-supplemented cows. Further studies are required to understand the mechanisms through which GCE influences the metabolism of cows to improve milk production performance. The effect of dietary GCE on improved milk production efficiency resulted in a lower CFP for milk when modelled in an LCA simulation. Thus, this study demonstrated that feeding GCE could be a viable nutritional solution for improving sustainable dairy production in grazing systems.

## Figures and Tables

**Table 1:** Chemical composition of the pasture, rolled corn, hay and concentrate fed to early- to mid-lactation cows in Trial 1.

Chemical Composition	Pasture	Rolled Corn	Hay	Concentrates
Dry matter (DM; %)	16.6	85.3	80.0	88.0
Crude protein (% DM)	22.0	8.10	10.4	16.1
Crude fat (% DM)	4.17	3.44	2.35	3.90
Neutral detergent fibre (% DM)	44.8	12.2	63.1	23.2
Acid detergent fibre (% DM)	24.8	4.90	40.2	11.4
Starch (% DM)	3.31	72.2	1.80	40.3
Metabolisable energy (Mcal/kg DM)	2.61	3.37	2.13	2.70
Net energy for lactation (Mcal/kg DM)	1.56	1.98	1.30	1.61
Calcium (% DM)	0.73	-	0.34	0.81
Phosphorous (% DM)	0.29	0.18	0.27	0.57
Magnesium (% DM)	0.29	0.09	0.15	0.29
Potassium (% DM)	2.53	0.28	2.26	0.93

**Table 2:** Chemical composition of the pasture, silage and concentrate fed to late-lactation cows in Trial 1.

Chemical Composition	Pasture	Ryegrass + Barley Silage	Concentrates
Dry matter (%)	20.2	30.5	88.4
Crude protein (% DM)	21.9	12.5	12.9
Crude fat (% DM)	4.19	3.63	1.79
Neutral detergent fibre (% DM)	45.0	53.2	19.5
Acid detergent fibre (% DM)	28.2	35.3	7.85
Metabolisable energy (Mcal/kg DM)	2.55	2.22	3.05
Net energy for lactation (Mcal/kg DM)	1.62	1.51	1.81
Calcium (% DM)	0.63	0.47	2.12
Phosphorous (% DM)	0.34	0.25	0.42
Magnesium (% DM)	0.27	0.18	0.52
Potassium (% DM)	2.74	1.50	0.82

**Table 3:** Effect of garlic and citrus extract supplement on milk yield and composition, feed intake and the feed efficiency of early- to mid-lactation cows in Trial 1.

Parameter	Treatment ^1^			*p*-Value		
	CTRL	GCE	SEM	Treatment	Week	Treatment × Week
Milk yield (kg/d)	25.4	28.0	0.43	<0.001	<0.001	0.231
ECM ^2^ (kg/d)	24.5	27.3	0.43	<0.001	<0.001	0.166
Protein (%)	3.49	3.47	0.02	0.619	0.063	0.097
Fat (%)	3.67	3.76	0.05	0.175	<0.001	0.439
Protein yield (kg/d)	0.88	0.97	0.02	<0.001	<0.001	0.037
Fat yield (kg/d)	0.92	1.05	0.02	<0.001	<0.001	0.361
Urea (mg/L)	372	367	4.43	0.428	<0.001	0.335
Somatic cell count (×1000 cells/mL)	41.9	36.1	6.51	0.550	0.161	0.902
Feed intake and efficiency						
Estimated DMI ^3^ (kg/d)	18.4	19.9	0.20	<0.001	<0.001	0.221
Milk yield/DMI	1.37	1.40	0.01	0.057	<0.001	0.329
ECM/DMI	1.32	1.36	0.01	0.001	<0.001	0.191

^1^ CTRL, control; GCE, garlic and citrus extract supplement. ^2^ ECM, energy-corrected milk. ^3^ DMI, dry matter intake.

**Table 4:** Effect of garlic and citrus extract supplement on milk yield and composition, feed intake and the feed efficiency of late-lactation cows in Trial 2.

Parameters	Treatment ^1^		SEM	*p*-Value		
	CTRL	GCE		Treatment	Week	Treatment × Week
Milk yield (kg/d)	14.9	19.6	0.21	<0.001	<0.001	<0.001
ECM ^2^ (kg/d)	15.5	20.7	0.27	<0.001	<0.001	<0.001
Protein (%)	4.04	4.03	0.03	0.894	<0.001	0.414
Fat (%)	4.25	4.20	0.07	0.674	<0.001	0.816
Protein yield (kg/d)	0.59	0.79	0.01	<0.001	<0.001	<0.001
Fat yield (kg/d)	0.60	0.81	0.01	<0.0001	0.006	<0.001
Urea (mg/L)	363	363	5.49	0.868	<0.001	0.181
Somatic cell count (×1000 cells/mL)	138	104	18.4	0.193	<0.001	0.447
Feed efficiency						
Estimated DMI ^3^ (kg/d)	15.0	17.3	0.14	<0.001	<0.001	<0.001
Milk yield/DMI	0.97	1.13	0.01	<0.001	<0.001	<0.001
ECM/DMI	1.01	1.19	0.01	<0.001	<0.001	<0.001

^1^ CTRL, control; GCE, garlic and citrus extract supplement. ^2^ ECM, energy-corrected milk. ^3^ DMI, dry matter intake.

**Table 5:** Effect of feeding garlic and citrus extract supplement (GCE) on greenhouse gas emission intensity by the source of emissions during the entire lactating cycle of dairy cows as simulated using baseline (control) and GCE scenarios in a life cycle assessment model.

Emission Sources (g CO_2_-eq/kg ECM ^1^)	CTRL-Baseline	GCE Scenario	% Change
Enteric fermentation CH_4_	1026	940	−8.38%
Manure CH_4_	26.9	24.7	−8.18%
Manure N_2_O	34.4	30.4	−11.6%
Feed N_2_O	173	157	−9.25%
Feed CO_2_	206	188	−8.74%
Feed LUC ^2^ CO_2_	0.33	0.31	−6.06%
Direct energy use CO_2_	70.8	70.8	0.00%
Indirect energy use CO_2_	10.8	9.41	−12.9%

^1^ ECM, energy-corrected milk. ^2^ LUC, land-use change.

## Data Availability

The data presented in this study are available from the corresponding author upon request.

## References

[1] Guerrero López A. (2019). ODEPA Boletín Sector Lácteo: Estadísticas de Comercio Exterior.

[2] Rojas Cofré C., Cáceres L., Tapia Cruz B. (2022). Análisis de Los Resultados Del VIII Censo Agropecuario y Forestal.

[3] Alfaro M., Salazar F. (2005). Ganaderia Y Contaminacion Difusa, Implicancias Para El Sur De Chile Volver a: Sustentabilidad. Agric. Técnica.

[4] Alfaro M., Salazar F., Iraira S., Teuber N., Villarroel D., Ramírez L. (2008). Pérdidas de Nitrógeno, Fósforo y Potasio de Un Sistema Pastoril Con Distinta Carga Animal En Un Suelo Volcánico. Chil. J. Agric. Res..

[5] Núñez P.A., Demanet R., Misselbrook T.H., Alfaro M., de la Luz Mora M. (2010). Pérdidas de Nitrógeno Bajo Diferentes Frecuencias e Intensidades de Pastoreo En Un Suelo Volcánico Del Sur de Chile. Chil. J. Agric. Res..

[6] Pulido R.G., Muñoz R., Jara C., Balocchi O.A., Smulders J.P., Wittwer F., Orellana P., O’Donovan M. (2010). The Effect of Pasture Allowance and Concentrate Supplementation Type on Milk Production Performance and Dry Matter Intake of Autumn-Calving Dairy Cows in Early Lactation. Livest. Sci..

[7] Keim J.P., Valderrama X., Alomar D., Lopez I.F. (2013). In Situ Rumen Degradation Kinetics as Affected by Type of Pasture and Date of Harvest. Sci. Agric..

[8] VandeHaar M.J., St-Pierre N. (2006). Major Advances in Nutrition: Relevance to the Sustainability of the Dairy Industry. J. Dairy Sci..

[9] Gerber P., Vellinga T., Opio C., Steinfeld H. (2011). Productivity Gains and Greenhouse Gas Emissions Intensity in Dairy Systems. Livest. Sci..

[10] Rivas M.C.B., Palacios Riocerezo C., Dominguez Vara I.A., Gonzalez Ronquillo M., Radic Schilling S. (2019). Production, Processing, Commercialization and Analysis of Costumer Preferences of Sheep Cheese in Chile. Milk Production, Processing and Marketing.

[11] Vrancken H., Suenkel M., Hargreaves P.R., Chew L., Towers E. (2019). Reduction of Enteric Methane Emission in a Commercial Dairy Farm by a Novel Feed Supplement. Open J. Anim. Sci..

[12] Ahmed E., Fukuma N., Hanada M., Nishida T. (2021). The Efficacy of Plant-Based Bioactives Supplementation to Different Proportion of Concentrate Diets on Methane Production and Rumen Fermentation Characteristics in Vitro. Animals.

[13] Bitsie B., Osorio A.M., Henry D.D., Silva B.C., Godoi L.A., Supapong C., Brand T., Schoonmaker J.P. (2022). Enteric Methane Emissions, Growth, and Carcass Characteristics of Feedlot Steers Fed a Garlic and Citrus Based Feed Additive in Diets with Three Different Forage Concentrations. J. Anim. Sci..

[14] Khurana R., Brand T., Tapio I., Bayat A.R. (2023). Effect of a Garlic and Citrus Extract Supplement on Performance, Rumen Fermentation, Methane Production, and Rumen Microbiome of Dairy Cows. J. Dairy Sci..

[15] Eger M., Graz M., Riede S., Breves G. (2018). Application of Mootral^TM^ Reduces Methane Production by Altering the Archaea Community in the Rumen Simulation Technique. Front. Microbiol..

[16] (2001). Nutrient Requirements of Dairy Cattle.

[17] Goering H.K., Van Soest P.J. (1970). Forage Fiber Analysis: Apparatus, Reagents, Procedures and Some Applications. USDA-ARS Agricultural Handbook 379.

[18] Van Soest P.J., Robertson J.B., Lewis B.A. (1991). Methods for Dietary Fiber, Neutral Detergent Fiber, and Nonstarch Polysaccharides in Relation to Animal Nutrition. J. Dairy Sci..

[19] The Association of Official Analytical Chemists (2000). AOAC Official Methods of Analysis.

[20] Weiss W.P. (1998). Estimating the Available Energy Content of Feeds for Dairy Cattle. J. Dairy Sci..

[21] (2013). Milk and Liquid Milk Products, Guidelines for the Application of Mid-Infrared Spectrometry.

[22] (2009). Milk-Definition and Evaluation of the Overall Accuracy of Alternative Methods of Milk Analysis—Part 2: Calibration and Quality Control in the Dairy Laboratory.

[23] (2006). Milk-Enumeration of Somatic Cells—Part 2: Guidance on the Operation of Fluoro-Opto-Electronic Counters, 2nd Ed.

[24] Souza M.C., Oliveira A.S., Araújo C.V., Brito A.F., Teixeira R.M.A., Moares E.H.B.K., Moura D.C. (2014). Short Communication: Prediction of Intake in Dairy Cows under Tropical Conditions. J. Dairy Sci..

[25] Engelke S.W., Daş G., Derno M., Tuchscherer A., Berg W., Kuhla B., Metges C.C. (2018). Milk Fatty Acids Estimated by Mid-Infrared Spectroscopy and Milk Yield Can Predict Methane Emissions in Dairy Cows. Agron. Sustain. Dev..

[26] Western Canadian Dairy Herd Improvement Services Persistency of Milk Production—Info Sheet. http://agromedia.ca/ADM_Articles/content/DHI_persist.pdf.

[27] MacLeod M.J., Vellinga T., Opio C., Falcucci A., Tempio G., Henderson B., Makkar H., Mottet A., Robinson T., Steinfeld H. (2018). Invited Review: A Position on the Global Livestock Environmental Assessment Model (GLEAM). Animal.

[28] Ross S.A., Topp C.F.E., Ennos R.A., Chagunda M.G.G. (2017). Relative Emissions Intensity of Dairy Production Systems: Employing Different Functional Units in Life-Cycle Assessment. Animal.

[29] Prayitno C.H., Suwarno, Susanto A., Jayanegara A. (2016). Effect of Garlic Extract and Organic Mineral Supplementation on Feed Intake, Digestibility and Milk Yield of Lactating Dairy Cows. Asian J. Anim. Sci..

[30] Balcells J., Aris A., Serrano A., Seradj A.R., Crespo J., Devant M. (2012). Effects of an Extract of Plant Flavonoids (Bioflavex) on Rumen Fermentation and Performance in Heifers Fed High-Concentrate Diets. J. Anim. Sci..

[31] Ding H., Ao C., Zhang X. (2023). Potential Use of Garlic Products in Ruminant Feeding: A Review. Anim. Nutr..

[32] Yu S., Li L., Zhao H., Zhang S., Tu Y., Liu M., Zhao Y., Jiang L. (2023). Dietary Citrus Flavonoid Extract Improves Lactational Performance through Modulating Rumen Microbiome and Metabolites in Dairy Cows. Food Funct..

[33] Seymour W.M., Campbell D.R., Johnson Z.B. (2005). Relationships between Rumen Volatile Fatty Acid Concentrations and Milk Production in Dairy Cows: A Literature Study. Anim. Feed Sci. Technol..

[34] Olagaray K.E., Bradford B.J. (2019). Plant Flavonoids to Improve Productivity of Ruminants—A Review. Anim. Feed Sci. Technol..

[35] Sharma N., Singh N.K., Singh O.P., Pandey V., Verma P.K. (2011). Oxidative Stress and Antioxidant Status during Transition Period in Dairy Cows. Asian-Aust. J. Anim. Sci..

[36] Petit H.V. (2009). Antioxidants and Dairy Production: The Example of Flax. Rev. Bras. Zootec..

[37] Boushehri M., Sadeghi A.A., Chamani M., Aminafshar M. (2021). Effects of Antioxidants and Prebiotics as Vegetable Pellet Feed on Production Performance, Hematological Parameters and Colostrum Immunoglobulin Content in Transition Dairy Cows. Ital. J. Anim. Sci..

[38] Tedesco D., Tava A., Galletti S., Tameni M., Varisco G., Costa A., Steidler S. (2004). Effects of Silymarin, a Natural Hepatoprotector, in Periparturient Dairy Cows. J. Dairy Sci..

[39] Koloi S., Pathak K., Behera R., Mandal D.K., Karunakaran M., Duta T.K., Mandal A. (2018). Factors Affecting the Persistency of Milk Production in Jersey Crossbred Cattle. J. Dairy Vet. Anim. Res..

[40] Gholipour A., Foroozandeh Shahraki A.D., Tabeidian S.A., Nasrollahi S.M., Yang W.Z. (2016). The Effects of Increasing Garlic Powder and Monensin Supplementation on Feed Intake, Nutrient Digestibility, Growth Performance and Blood Parameters of Growing Calves. J. Anim. Physiol. Anim. Nutr..

[41] Rossi G., Schiavon S., Lomolino G., Cipolat-Gotet C., Simonetto A., Bittante G., Tagliapietra F. (2018). Garlic (*Allium sativum* L.) Fed to Dairy Cows Does Not Modify the Cheese-Making Properties of Milk but Affects the Color, Texture, and Flavor of Ripened Cheese. J. Dairy Sci..

[42] Ghosh S., Mehla R.K., Sirohi S.K., Roy B. (2010). The Effect of Dietary Garlic Supplementation on Body Weight Gain, Feed Intake, Feed Conversion Efficiency, Faecal Score, Faecal Coliform Count and Feeding Cost in Crossbred Dairy Calves. Trop. Anim. Health Prod..

[43] Velarde-Guillén J., Arndt C., Gómez C.A. (2022). Carbon Footprint in Latin American Dairy Systems. Trop. Anim. Health Prod..

[44] Celis Hidalgo J.E., Allende R., Mardones L. (2013). Empiric Approximation for the Carbon Footprint Determination from a Semi Intensive Dairy Farm in Chile. Vet. Sci. Anim. Husb..

[45] Gerber P., Vellinga T., Opio C., Henderson B., Steinfeld H. (2010). Greenhouse Gas Emissions from the Dairy Sector—A Life Cycle Assessment.

[46] O’Brien D., Brennan P., Humphreys J., Ruane E., Shalloo L. (2014). An Appraisal of Carbon Footprint of Milk from Commercial Grass-Based Dairy Farms in Ireland According to a Certified Life Cycle Assessment Methodology. Int. J. Life Cycle Assess..

[47] Yan M.J., Humphreys J., Holden N.M. (2013). The Carbon Footprint of Pasture-Based Milk Production: Can White Clover Make a Difference?. J. Dairy Sci..

[48] O’Brien D., Hennessy T., Moran B., Shalloo L. (2015). Relating the Carbon Footprint of Milk from Irish Dairy Farms to Economic Performance. J. Dairy Sci..

[49] Gollnow S., Lundie S., Moore A.D., McLaren J., van Buuren N., Stahle P., Christie K., Thylmann D., Rehl T. (2014). Carbon Footprint of Milk Production from Dairy Cows in Australia. Int. Dairy J..

[50] Christie K.M., Gourley C.J.P., Rawnsley R.P., Eckard R.J., Awty I.M. (2012). Whole-Farm Systems Analysis of Australian Dairy Farm Greenhouse Gas Emissions. Anim. Prod. Sci..

[51] Ledgard S.F., Falconer S.J., Abercrombie R., Philip G., Hill J.P. (2020). Temporal, Spatial, and Management Variability in the Carbon Footprint of New Zealand Milk. J. Dairy Sci..

[52] Flysjö A., Henriksson M., Cederberg C., Ledgard S., Englund J.E. (2011). The Impact of Various Parameters on the Carbon Footprint of Milk Production in New Zealand and Sweden. Agric. Syst..

[53] Gerber P., Opio C. (2010). Greenhouse Gas Emission from Ruminant Supply Chains: A Global Life Cycle Assessment.

[54] Gerber P.J., Steinfeld H., Henderson B., Mottet A., Opio C., Dijkman J., Falcucci A., Tempio G. (2013). Tackling Climate Change through Livestock: A Global Assessment of Emissions and Mitigation Opportunities.

[55] Beauchemin K.A., Ungerfeld E.M., Abdalla A.L., Alvarez C., Arndt C., Becquet P., Benchaar C., Berndt A., Mauricio R.M., McAllister T.A. (2022). Invited Review: Current Enteric Methane Mitigation Options. J. Dairy Sci..

